# 1,25(OH)_2_D_3_ Alters Growth Plate Maturation and Bone Architecture in Young Rats with Normal Renal Function

**DOI:** 10.1371/journal.pone.0020772

**Published:** 2011-06-13

**Authors:** Anna Idelevich, Michael Kerschnitzki, Ron Shahar, Efrat Monsonego-Ornan

**Affiliations:** 1 Institute of Biochemistry, Food Science and Nutrition, Hebrew University of Jerusalem, Rehovot, Israel; 2 Koret School of Veterinary Medicine, The Robert H. Smith Faculty of Agriculture, Food and Environment, Hebrew University of Jerusalem, Rehovot, Israel; 3 Department of Biomaterials, Max Planck Institute of Colloids and Interfaces, Potsdam, Germany; University of Western Ontario, Canada

## Abstract

Whereas detrimental effects of vitamin D deficiency are known over century, the effects of vitamin D receptor activation by 1,25(OH)_2_D_3_, the principal hormonal form of vitamin D, on the growing bone and its growth plate are less clear. Currently, 1,25(OH)_2_D_3_ is used in pediatric patients with chronic kidney disease and mineral and bone disorder (CKD-MBD) and is strongly associated with growth retardation. Here, we investigate the effect of 1,25(OH)_2_D_3_ treatment on bone development in normal young rats, unrelated to renal insufficiency. Young rats received daily i.p. injections of 1 µg/kg 1,25(OH)_2_D_3_ for one week, or intermittent 3 µg/kg 1,25(OH)_2_D_3_ for one month. Histological analysis revealed narrower tibial growth plates, predominantly in the hypertrophic zone of 1,25(OH)_2_D_3_-treated animals in both experimental protocols. This phenotype was supported by narrower distribution of aggrecan, collagens II and X mRNA, shown by *in situ* hybridization. Concomitant with altered chondrocyte maturation, 1,25(OH)_2_D_3_ increased chondrocyte proliferation and apoptosis in terminal hypertrophic cells. *In vitro* treatment of the chondrocytic cell line ATDC5 with 1,25(OH)_2_D_3_ lowered differentiation and increased proliferation dose and time-dependently. Micro-CT analysis of femurs from 1-week 1,25(OH)_2_D_3_-treated group revealed reduced cortical thickness, elevated cortical porosity, and higher trabecular number and thickness. 1-month administration resulted in a similar cortical phenotype but without effect on trabecular bone. Evaluation of fluorochrome binding with confocal microscopy revealed inhibiting effects of 1,25(OH)_2_D_3_ on intracortical bone formation. This study shows negative effects of 1,25(OH)_2_D_3_ on growth plate and bone which may contribute to the exacerbation of MBD in the CKD pediatric patients.

## Introduction

During childhood, the skeleton undergoes rapid structural adaptations to the growing body demands, manifested by changes in the bone mineral composition, cortical and trabecular architecture and altered mechanical properties. Bone mass acquisition is orchestrated by a variety of factors ranging from genetic determinants and nutritional influences, to the hormonal balance, including classical regulation of mineral homeostasis by vitamin D [1], [2]. Vitamin D is obtained from dietary sources and is synthesized in the skin by photoconversion of 7-dehydrocholesterol to vitamin D3, which subsequently undergoes two major modifications. First, it is metabolized in the liver to produce the circulating form 25-hydroxyvitamin D_3_ (25(OH)D_3_, calcidiol), which is later converted in the kidney and other tissues including bone by 1α-hydroxylase to generate the active form 1,25-hydroxyvitamin D_3_ (1,25(OH)_2_D_3_, calcitriol). 1,25(OH)_2_D_3_ is the principal hormonal form of vitamin D which binds to vitamin D receptor (VDR), exerting a wide range of biological responses [1], [2].

Whereas detrimental effects of vitamin D deficiency on bone growth have been known over a century, the direct effects of VDR activation on the growth plate are still a matter of investigation. Analysis of transgenic VDR or 1α-hydroxylase null mice revealed phenotypic abnormalities characteristic of vitamin D-dependent rickets, with decreased bone mineralization, growth retardation and aberrant growth plate development after weaning. Impaired apoptosis of hypertrophic chondrocytes, with ordinary proliferation, was demonstrated to cause the significant widening and disorganization of the epiphyseal growth plates [3]. These phenotypes could largely be corrected by rescue supplementation of dietary calcium and phosphate, suggesting a primary systemic role of 1,25(OH)_2_D_3_ in intestinal calcium absorption [4], [5]. In contrast, transplantation of VDR null bone into wild-type mice resulted in increased bone mass and *vice versa*, transplantation of wild-type bone into VDR null mice developed osteopenia, implying a direct inhibitory role of 1,25(OH)_2_D_3_ in osteogenesis [6]. To support these data, conditional VDR inactivation in growth plate chondrocytes has been shown to reduce vascular invasion and osteoclast numbers leading to increased trabecular bone mass [7]. Two additional transgenic mice models with chondrocytes-specific 1α-hydroxylase ablation or overexpression, revealed a “mirror image” growth plate phenotype with increased hypertrophic zone width, reduced osteoclastogenesis and delayed angiogenesis in the former, while decreased hypertrophic zone width, enhanced osteoclastogenesis and induced angiogenesis in the later, further supporting the paracrine role 1,25(OH)_2_D_3_ in the process of endochondral ossification [8].

Besides its physiological role, the pharmacological influence of 1,25(OH)_2_D_3_ on bone development in pediatric patients is not fully understood. In the clinical settings, administration of 1,25(OH)_2_D_3_ is currently used as a standard of care treatment combating both 1,25(OH)_2_D_3_ deficiency and secondary hyperparathyroidism in children with chronic kidney disease-mineral and bone disorder (CKD-MBD) [9]. In the pediatric patients, diminished renal function is commonly accompanied by a disturbed bone metabolism, reduced linear growth and the presence of ectopic soft tissue calcifications [9], [10]. Despite its widespread use, accumulating evidence points towards severe side effects and chronic toxicity associated with active vitamin D treatment [10], [11], [12]. Observational studies suggest that chronic use of 1,25(OH)_2_D_3_ is strongly associated with suppression of PTH and development of adynamic bone disease (ABD), characterized by low bone turnover and diminished osteoblast and osteoclast activities, in children with renal insufficiency [9], [10]. Clinical studies performed in children on peritoneal dialysis treated for 12 months with intermittent calcitriol therapy and calcium salts as phosphate binders, demonstrated diminished linear growth and induction of ABD, accompanied by significant reduction in PTH and episodes of hypercalcemia [13], [14], [15]. Linking bone and cardiovascular health, both ABD and 1,25(OH)_2_D_3_ were reported to increase the occurrence of vascular calcification (VC), known as a leading factor of mortality in CKD patients [11], [16]. The risk for cardiovascular complications exhibits a biphasic curve, with highest risk involving vitamin D deficiency, uncontrolled hyperparathyroidism and high bone turnover, on one hand, and vitamin D-induced low PTH and ABD on the other hand [11], [17].

In attempt to understand the role of vitamin D in CKD-MBD state, several studies examined the effect of 1,25(OH)_2_D_3_ treatment on growth plate and bone development in renal failure animal models. Daily or intermittent treatment with 1,25(OH)_2_D_3_ for a period of 4 weeks of 5/6 nephrectomized weanling rats with secondary hyperparathyroidism was shown to increase the markers of growth plate chondrocytes proliferation [18]. Short-term treatment of uremic rats with 1,25(OH)_2_D_3_ resulted in a reduced number of chondro-osseous junction chondroclasts compared to uremic control [19]. Examination of bone micro-architecture in nephrectomized growing rats revealed significantly altered bone parameters, including increased trabecular thickness, reduced cortical thickness and increased porosity, which were aggravated by daily administration of 1,25(OH)_2_D_3_ [20]. Treatment with calcimimetic VDR analogues in a similar uremic rat model were also demonstrated to affect bone formation reducing osteoid surface and increasing bone resorption [21]. Collectively, these studies suggest that active vitamin D influences growth plate and bone formation in the young skeleton and may contribute to the diminished growth in CKD-MBD children.

At present, limited data is available on the direct effects of 1,25(OH)_2_D_3_ in bone tissue under physiological conditions, separate from renal insufficiency. In the current study we investigate the influence of continuous 1-week and intermittent 1-month administration of 1,25(OH)_2_D_3_ on growth plate and bone development in normal young rats.

## Materials and Methods

### Cell culture and treatment with 1,25(OH)_2_D_3_

ATDC5 cell line [22] was cultured in DMEM/F-12 medium (Bet-Haemek) supplemented with 5% FBS, 1% Insulin-transferrin-sodium selenite (Sigma-Aldrich), penicillin (100 units/mL) / streptomycin (0.1 mg/mL), and 4 mM L-Glutamine. 1,25(OH)_2_D_3_ (Sigma-Aldrich) was dissolved in ethanol and stored as 41.6 µg/mL (100 µM ) stock at −20C° until use. For differentiation experiments, cells were seeded at initial density of 5×10^4^ cells / well in 12 well plates and treated the following day with 1–30 nM 1,25(OH)_2_D_3_ dissolved in normal medium or same volume of ethanol dissolved in medium, as control. Cells were treated twice 1-week for a period of 1, 7, 14, and 21 days [22]. Cells were observed under inverted Nikon Eclipse TS100 microscope equipped with DS-F1camera control unit.

### *In vitro* proliferation and differentiation of ATDC5

Throughout ATDC5 differentiation and treatment with 1,25(OH)_2_D_3_ experiment cell media were collected and stored at −20C°. Staining with Alizarin Red, Alcian Blue, and Alkaline Phosphatase (ALP) and MMP zymography were performed as described previously [22], [23], [24]. Secreted ALP activity was measured by adding 100 µL of 0.5 mM pNPP substrate dissolved in basic buffer: 0.1 M Tris-HCl, 0.1 M NaCl, 0.05 M MgCl_2_, pH 9.5, to 100 µL of cell medium in 96 well plate and incubating 30 min at RT, protected from light. Hydrolyzed pNPP was assessed by absorbance at 405 nm. For secreted GAG detection 100 µL of DMB solution (16 g 1,9-dimethylmethylene blue (DMB) dissolved in 55 mmol/L formic acid, pH 3.3) solution was added to 100 µL of cell medium in 96 well plate, incubated 2 hrs at RT and binding complex absorbance was read at 595 nm (non-complexed signal). DMB-GAG complex formation was calculated. Bone Gla Protein (BGP, also known as osteocalcin) levels in cell media were identified by ELISA using Mouse Osteocalcin EIA kit (Biomedical Technologies), according to manufacturer's instructions. Metabolic activity was measured using XTT kit (Biological Industries) according to manufacturer's instructions.

### Gene quantification with real-time PCR

RNA was extracted using TRI-reagent (Sigma-Aldrich) according to manufacturer's instructions. 1 µg of total RNA was reversed transcribed using High Capacity cDNA kit (Applied Biosystems). cDNA was diluted 1∶25 and used for real-time PCR using Platinum SYBR Green qPCR SuperMix-UDG with ROX (Invitrogen) to measure gene expression using specific primers sets: Collagen typeII(f) GAACAGCATCGCCTACCTGG, Collagen typeII(r) TGTTTCGTGCAGCCATCCT; Sox9(f) GCATCTGCACAACGCGG, Sox9(r) CTCGTTCAGCAGCCTCCAG; Runx2(f) AGGCACAGACAGAAGCTTGATG, Runx2(r) GCGATCAGAGAACAAACTAGGTTTAGA; MMP2(f) ATTGACGCTGTGTATGAGGCC, MMP2(r) ACTCATTCCCTGCGAAGAACA; MMP9(f) AGCCCCTGCTCCTGGCTCTC, MMP9(r) CTGCCAGCTGGGTGTCCGTG; GAPDH(f) TGACGTGCCGCCTGGAGAAA, GAPDH(r) AGTGTAGCCCAAGATGCCCTT. Dissociation curves were ran following Real-Time PCR reactions to insure the detection of the desired amplicon and exclude the presence of contaminating products. Amplification was carried out using 7300 Fast Real-Time PCR System (Applied Biosystems). Gene expression was normalized to GAPDH and the data were analyzed using comparative 2^−ΔΔCt^ method [25].

### Animal experiments

The studies were approved by the Hebrew University of Jerusalem Animal Care and Use Committee, Ethics number: AG-09-12147-2. Male Sprague-Dawly rats (75 g, 4 weeks old) were purchased from Harlen Labratories (Rehovot, Israel) and fed a normal chow diet. Two experimental schedules were performed, one week experiment and one month experiment. For a one week experiment rats were randomly allocated into two groups: (1) received i.p. injections of 2.4% (v/v) ethanol in saline vehicle, daily for 7 days (n = 6); (2) received i.p. injections of 1 µg/kg 1,25(OH)_2_D_3_ in saline, daily for 7 days (n = 6). For a one month experiment: (1) received i.p. injections of 2.4% (v/v) ethanol in saline vehicle three times a week for 30 days (n = 6); (2) received i.p. injections of 3 µg/kg 1,25(OH)_2_D_3_ in saline, three times a week for 30 days (n = 6). The total volume of injection was 100 µL per 100 mg of rat body weight. In the clinical settings, secondary hyperparathyroidism associated with moderate-to-severe CKD-MBD in pediatric patients is managed with doses reaching 0.025 µg/kg daily (The Merck Manual). We have chosen to administer about 40 times higher dose due the following reasons. First, both of our experiments were designed to span a considerably shorter time-frame, in comparison to chronic 1,25(OH)_2_D_3_ treatment practiced in CKD-MBD patients, where drug and its effects may be accumulated over time. Second, during the allometric dose translation from animals to humans, in addition to body weight (BW), Km factor representing BW (kg) divided by body surface area (BSA, m^2^) of both species is commonly taken into account. BSA has been previously shown to correlate with several parameters including oxygen utilization, caloric expenditure, basal metabolism, blood volume, circulating plasma proteins, and renal function [26]. Thus, according to the Food and Drug administration recommendations, dose given to rats should be about 6.2 times higher than dose given to humans [26]. To accommodate these two notions we decided to administer a dose higher than normally given to pediatric CKD-MBD patients, which in our view serves a better representation of the clinical picture. Both groups in one week experiment also received i.p. injections of mineral binding fluorochromes, 40 mg/kg calcein on day 2 and 20 mg/kg alizarin complexon on day 5. At the end of the experiment the animals were anesthetized by Isoflurane and killed by cervical dislocation. Tibiae were removed, decalcified and embedded in paraffin blocks for histological analysis, femurs were frozen at −20C° until subsequent micro-CT analysis.

### Micro-CT

The region of proximal metaphysis to mid- diaphysis of all femurs , was scanned with a Skyscan 1174 X-ray computed microtomograph scanner (Skyscan, Aartselaar Belgium), equipped with a CCD detector. Images were obtained by 50 kV X-ray tube voltage and 800 µA current. Specimens from the 7-day calcitriol experiment were scanned using 0.25 mm aluminum filter, at 3500 ms exposure time, and at 13.8 pixel size resolution. For samples belonging to the 1-month calcitirol experiment 0.5 mm aluminum filter, 4000 ms exposure time and 13.8 pixel size resolution were used. For each specimen, a series of 900 projection images were obtained with a rotation step of 0.4°, averaging 2 frames, for a total 360° rotation. Flat field correction was performed at the beginning of each scan for a specific zoom and image format. A stack of 2D X-ray shadow projections was reconstructed to obtain images using NRecon software (Skyscan), and subjected to the morphometric analysis using CTAn software (Skyscan). During reconstruction, dynamic image range, post-alignment value, beam hardening and ring-artifact reduction were optimized for each experimental set. Analysis of the diaphyseal cortical region 200 slices, corresponding to 2.764 mm were chosen. Global greyscale threshold levels for the cortical region were between 100 and 255 were selected. For the trabecular region a total of 150 slices, corresponding to 2.073 mm were selected, and adaptive grayscale threshold levels between 40 (1-week experiment) and 60 (1-month experiment) to 255were used. Morphometric analysis was based on the 2-D and 3-D internal CTAn plug-ins. 3-D images (CTM file format) were constructed from cortical and trabecular regions of interest, utilizing Marching Cubes 33 algorithm in CTVol software [27].

### Histology and immunohistochemistry

Tibiae were fixed overnight in 4% paraformaldehyde (Sigma-Aldrich) in PBS at 4°C and subjected to 3 weeks of decalcification in 0.5 M EDTA, pH 7.4. Following dehydration in graded ethanol solutions and histoclear (Bar-Naor), tissue was embedded in Paraplast, cut into 5 µm sections and mounted on Superfrost slides (Thermo Scientific). For Safranin O staining, 0.1% Safranin O (Sigma) solution was used. Growth plate total width and areas width were measured using ImageJ software. For that, total growth plate was visually separated into two areas, based on morphological distinctions: 1. Proliferative zone, including columnar chondrocytes and intense orange (Safranin O stained) extracellular matrix; 2. Hypertrophic zone, including swollen mature, hypertrophic and terminally differentiated chondrocytes, characterized by low extracellular matrix volume. In each zone, 7 vertical (perpendicular to chondro-osseous junction) lines were drawn throughout the growth plate and width was calculated as an average of these 7 measurements. For immunohistochemistry, sections were deparaffinized in xylene and washed twice with ethanol. Endogenous peroxidase activity was blocked with 0.3% H_2_O_2_ (Merck) in methanol for 30 min. Sections were rehydrated through a graded series of ethanol solutions, rinsed in PBS+T and blocked with 3% goat serum in PBS+T for 1 hr. Specific antibodies were diluted in 3% goat serum in PBS+T and incubated for 1–2 hrs. For PCNA immunostaining a pair of primary mouse monoclonal anti-PCNA antibody 1∶200 (Dako) and secondary goat anti-mouse 1∶400 (Jackson ImmunoResearch) was used. HRP activity was measured using DAB substrate (Sigma-Aldrich). Staining was observed under Nikon Eclipse E400 microscope equipped with Olympus D71 camera and cell∧A software [28].

### Probe preparation and *in-situ* hybridization

BlueScript constructs containing Collagen type II, Aggrecan, Collagen type X were kindly provided by Dr. Eliezer Zeltzer. Sense and antisense digoxigenin-labeled RNA probes were transcribed using either T7 or T3 primers using DIG labeling kit (Roche). Hybridization of 5 µm paraffin-embedded sections and visualization with alkaline phosphatase–coupled anti-digoxigenin antibodies and indolylphosphate-nitroblue tetrazolium (BCIP/NBT) substrate were performed. Parallel sections were hybridized with antisense and sense probes. Briefly, sections were rehydrated in serial ethanol dilutions, fixed in 4% PFA, and treated with proteinase K (4 µg/ml in 0.2 M Tris-HCl, 5 mM EDTA, pH 7.5) for 20 min. After digestion, slides were rinsed, fixed again and acetylated in 0.1 M triethanolamine to reduce hydrogen bonding. RNA probes were dissolved to hybridization buffer (10 mM Tris pH 7.5, 600 mM NaCl, 1 mM EDTA, 0.25% SDS, 10% Dextran Sulfate (American Bioanalytical 50% solution), 1× Denhardt's, 200 µg/ml yeast tRNA (Gibco), 50% formamide) for a final concentration of 1 µg/mL. Following overnight hybridization at 65°C, slides were incubated with increasing concentrations of SSC to promote denaturing of nucleic acids and then digested with RNAse to eliminate unbound single stranded RNA. DIG-labeled probes were detected using a polyclonal anti-digoxigenin antibody attached to alkaline phosphatase (Roche) which produces color response when reacts with its substrate NBT+BCIP (Promega). Endogenous ALP was inhibited with Levamisole (Fluka). In all hybridizations, no signal was observed with sense probes which were used as negative controls [29].

### Terminal deoxynucleotidyl Transferase Biotin-dUTP Nick End Labeling (TUNEL) and tartarate-resistent acid phosphatase (TRAP)

TUNEL staining of the apoptotic cells was performed using *In Situ* Cell Death Detection Kit, POD (Roche), and DNA was counterstained with DAPI. Chondroclast invasion was assessed by TRAP staining kit (Sigma-Aldrich). Both assays were done according to manufacturer's instructions.

### Sample preparation and confocal laser scanning microscopy

Femurs from the mid-shaft were transversally sectioned with a low speed saw (IsoMet, Buehler GmbH, Düesseldorf, Germany) with an initial thickness of 200 µm. Samples were then polished by hand from both sides until a final thickness of 80 µm. For mechanical fixation during polishing, samples were embedded in acrylate (Sigma-Aldrich) and glued with double-sided tape to a polishing holder. Confocal laser scanning microscopy (CLSM) was performed using Leica DMI4000B equiped with Leica Application Suite software. Micrographs were taken using 10× and 43× objectives with a numerical aperture of 0.75. The excitation wavelength for the calcein was set to 488 nm, while the emission was measured at a range from 505 up to 530 nm. The excitation wavelength for the alizarine complexon was set to 532 nm, with measurement of the emission at a range from 660 up to 760 nm.

### Statistical analysis

All data are expressed as mean ± SD or SE. The significance of differences between groups was determined using JMP 8.0 Statistical Discovery Software (SAS Institute 2000) by one-way analysis of variance. Differences between groups were further evaluated by Tukey-Kramer HSD test. Differences were considered significant at P<0.05.

## Results

### 1,25(OH)_2_D_3_ reduces tibial growth plate width in young rats

**Figure 1:**
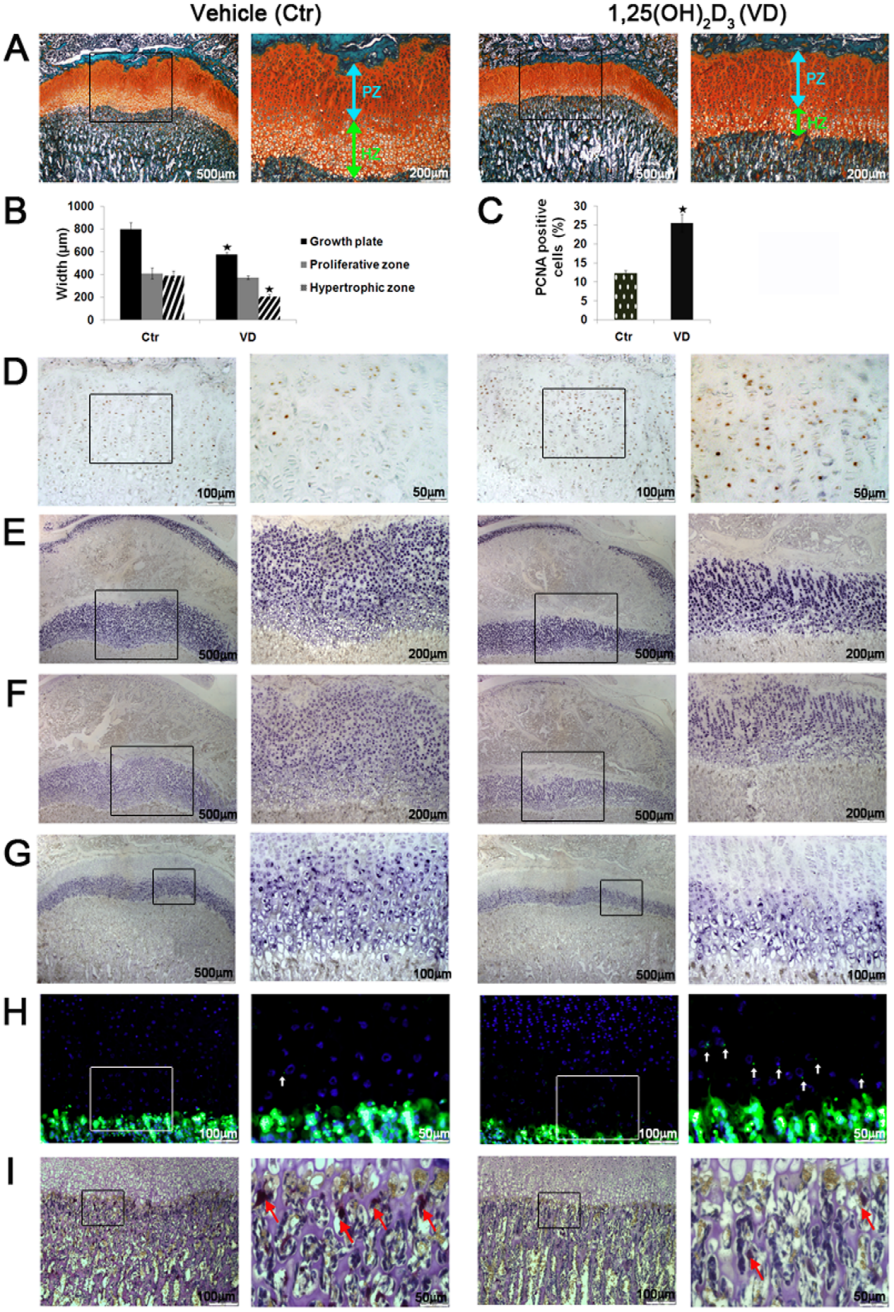
1-week continuous administration of 1,25(OH)_2_D_3_ induces chondrocyte proliferation and compresses chondrocytes maturation zone in young rats. 4 weeks old male Spague Dawley rats received daily i.p. injections of 1 µg/kg 1,25(OH)_2_D_3_ (n = 6, VD) or vehicle (n = 6, Ctr) for a period of 7 days. (A) Safranin O staining of proximal tibial growth plates. Arrows indicate reduced hypertrophic zone width in 1,25(OH)_2_D_3_-treated compared to the control group. (B) Width measurements of total growth plate, proliferative zone, and hypertrophic zone. (C) Percent of PCNA positive proliferating cells out of total proliferating cell count. (D) Immunhistochemistry of PCNA positive proliferating cells. (E) *In-situ* hybridization (*ISH*) of collagen type II mRNA. (F) *ISH* of aggrecan mRNA. (G) *ISH* of collagen type X mRNA. (H) TUNEL detection of apoptotic cells, indicated by white arrows. Cell nuclei were counterstained with DAPI. (I) TRAP staining for chondroclasts, indicated by arrows. In all studies, results are shown as means (n = 6) ± SD. * denotes p<0.05 comparing 1,25(OH)_2_D_3_-treated to control groups. Open squares in the images on the left represent areas magnified in the images on the right. Abbreviations: PZ-proliferative zone, HZ-hypertrophic zone.

**Figure 2:**
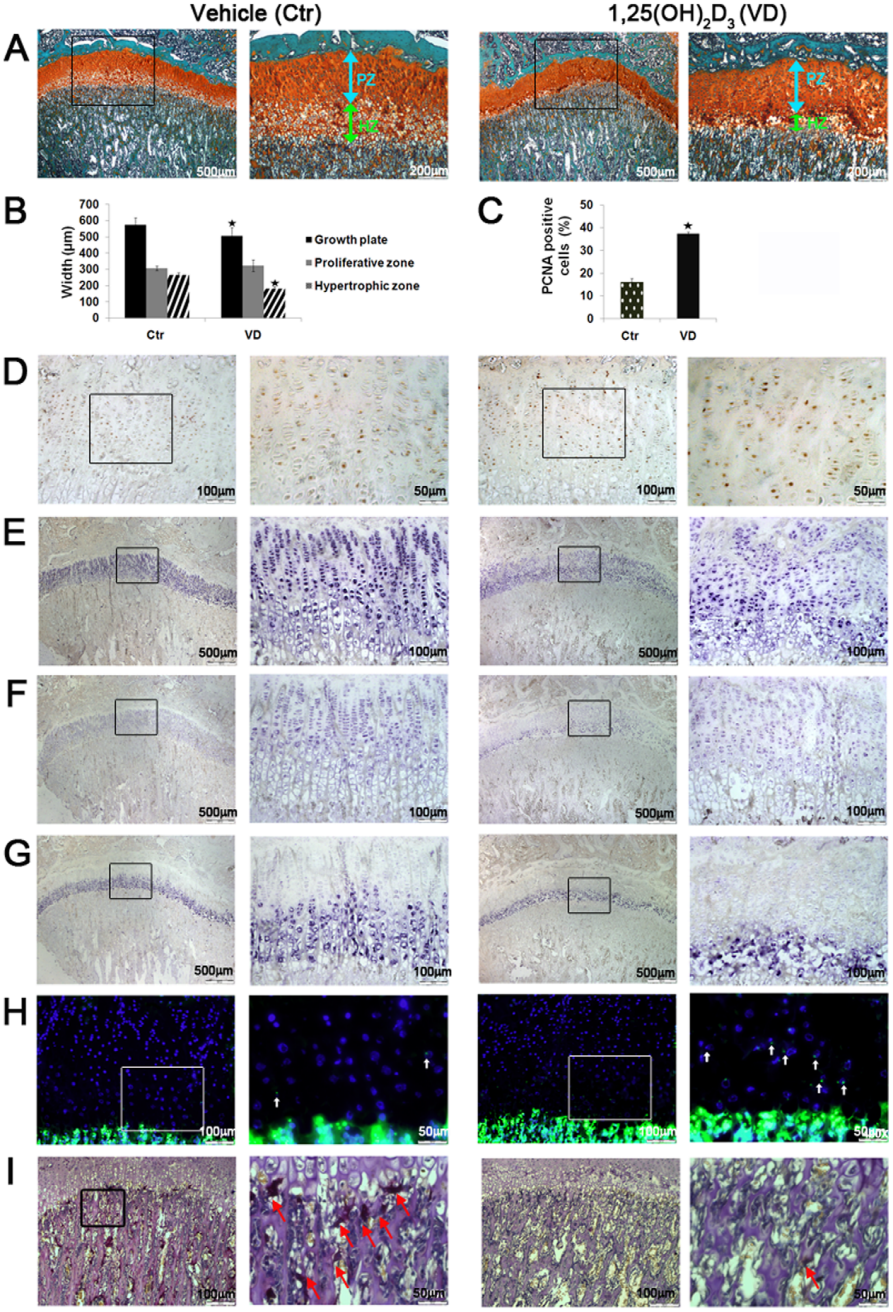
1-month intermittent administration of 1,25(OH)_2_D_3_ induces chondrocyte proliferation and compresses chondrocytes maturation zone in young rats. 4 weeks old male Spague Dawley rats received i.p. injections of 3 µg/kg 1,25(OH)_2_D_3_ (n = 6, VD) or vehicle (n = 6, Ctr) on alternating days, three times a week for a period of 30 days. (A) Safranin O staining of tibial proximal growth plates. Arrows indicate reduced hypertrophic zone width in 1,25(OH)_2_D_3_-treated compared to the control group. (B) Width measurements of total growth plate, proliferative zone, and hypertrophic zone. (C) Percent of PCNA positive proliferating cells out of total proliferating cell count. (D) Immunhistochemistry of PCNA positive proliferating cells. (E) *ISH* of collagen type II mRNA. (F) *ISH* of aggrecan mRNA. (G) *ISH* of collagen type X mRNA. (H) TUNEL detection of apoptotic cells, indicated by white arrows. Cell nuclei were counterstained with DAPI. (I) TRAP staining for chondroclasts, indicated by arrows. In all studies, results are shown as means (n = 6) ± SD. *denotes p<0.05 comparing 1,25(OH)_2_D_3_-treated to control group. Open squares in the images on the left represent areas magnified in the images on the right. Abbreviations: PZ-proliferative zone, HZ-hypertrophic zone.

Previously, VDR activation was shown to affect growth plate development [18], [19], and induce vascular calcification [30], in rats with chronic renal damage. To assess whether pharmacological administration of 1,25(OH)_2_D_3_ influences bone formation in young rats with normal renal function we established two regiments where 1,25(OH)_2_D_3_, was administered to young rats for a period of one week or one month. 1-week and 1-month treatment with 1,25(OH)_2_D_3_ induced vascular calcification, as evident by intense Alizarin Red staining of the aortae, compared to control (Fig S1A,B). Staining of tibiae with Safranin O (stain for cartilage) revealed marked reduction in the total growth plate width in both experiments (Fig. 1A, 2A). Measurement of individual zones showed that this effect is attributed predominantly to the narrowing of the hypertrophic zone of the growth plate, since the proliferative zone width remained unchanged (Fig. 1B, 2B). Hypertrophic zone width was reduced by 47% after 1-week 1,25(OH)_2_D_3_ treatment and 32% after 1-month treatment compared to the relevant controls. While continuous 1-week 1,25(OH)_2_D_3_ administration did not significantly alter the morphological appearance of chondrocytes, intermittent 1-month treatment resulted in disordered chondrocyte morphology, noticeably disrupting the columnar organization of the growth plate (Fig. 2A,E). Growth plate phenotypes were not manifested in significant tibial length differences (and final weight) between experimental groups, suggesting presence of compensating mechanisms which regulate longitudinal growth in this model.

### 1,25(OH)_2_D_3_ induces chondrocytes proliferation and reduces the expression of hypertrophic chondrocyte markers

Given the unchanged width of the proliferative zone, we subsequently tested whether 1,25(OH)_2_D_3_ altered the number of proliferating cells. Both 1,25(OH)_2_D_3_ treatment groups showed elevated ratio of PCNA positive cells out of the total population of proliferating cells, increasing by 107% after 1-week treatment, and by 131% after 1-month treatment (Fig. 1C,D, 2C,D). The count of total proliferating cells did not significantly vary between groups. Narrowing of the growth plate may stem from a number of mechanisms including hindered or accelerated chondrocyte maturation, elevated apoptosis, enhanced chondroclastogenesis and osteogenesis [31], [32]. To investigate this question, we fist examined the expression of several chondrocyte differentiation markers. *In situ* hybridization (*ISH*) analysis using specific probes for aggrecan and collagen type II, major structural components of the growth plate cartilage, demonstrated their compressed distribution in the 1,25(OH)_2_D_3_-treated groups compared to controls (Fig. 1E,F, 2E,F). As aggrecan and collagen type II are normally expressed in all growth plate zones, excluding the terminally differentiated chondrocytes [33], the effect observed is presumably due to the marked narrowing of the hypertrophic areas. Whereas 1-week 1,25(OH)_2_D_3_ treatment showed equal intensities of aggrecan and collagen type II mRNA staining throughout the growth plate, 1-month 1,25(OH)_2_D_3_ treatment resulted in weaker staining in the proliferative zone and more intense staining in the hypertrophic zone chondrocytes. In this regard, considering the observation of disrupted columnar organization it is plausible to assume that chondrogenesis is affected not only in the mature hypertrophic zone, but also in the earlier stages of chondrocyte proliferation during 1-month 1,25(OH)_2_D_3_ treatment. We speculate that a combined notion of increased PCNA staining and delayed escalation in collagen type II, as well as aggrecan expression, points toward a 1,25(OH)_2_D_3_-meidated disruption in the differentiation balance, favoring chondrocytes proliferation over maturation. *ISH* using probe for collagen type X, a specific marker for hypertrophic chondrocytes, supported these results, by showing a significantly reduced area of collagen type X expression in 1,25(OH)_2_D_3_ –treated compared to control tibiae in both groups (Fig. 1G, 2G). These data suggest that both continuous or intermittent treatment with 1,25(OH)_2_D_3_ augments proliferation and alters hypertrophic maturation of growth plates chondrocytes.

### 1,25(OH)_2_D_3_ induces hypertrophic chondrocyte apoptosis and suppresses chondroclast invasion

Following the observation of compressed growth plate and diminished chondrocyte hypertrophy we investigated plausible mechanisms which might contribute to the development of this phenotype. TUNEL, labeling DNA strand nicks, showed that both 1-week and 1-month 1,25(OH)_2_D_3_ treatment significantly increases the number of apoptotic cells in the hypertrophic zone compared to the respective control groups (Fig. 1H, 2H). All the TUNEL-positive cells were present in the maturation and hypertrophic layers of the growth plate. Surprisingly, the invasion of chondroclasts, associated with cartilage resorption, was markedly diminished by both 1-week and 1-month 1,25(OH)_2_D_3_ –treatment regiments, as shown by TRAP staining (Fig. 1I, 2I). These results suggest that enhanced apoptotic death, but not enhanced chondroclastogenesis may account for the reduced width of the growth plate hypertrophic zone.

### 1,25(OH)_2_D_3_ increases proliferation, while inhibiting chondrocytic differentiation of ATDC5 cells

**Figure 3:**
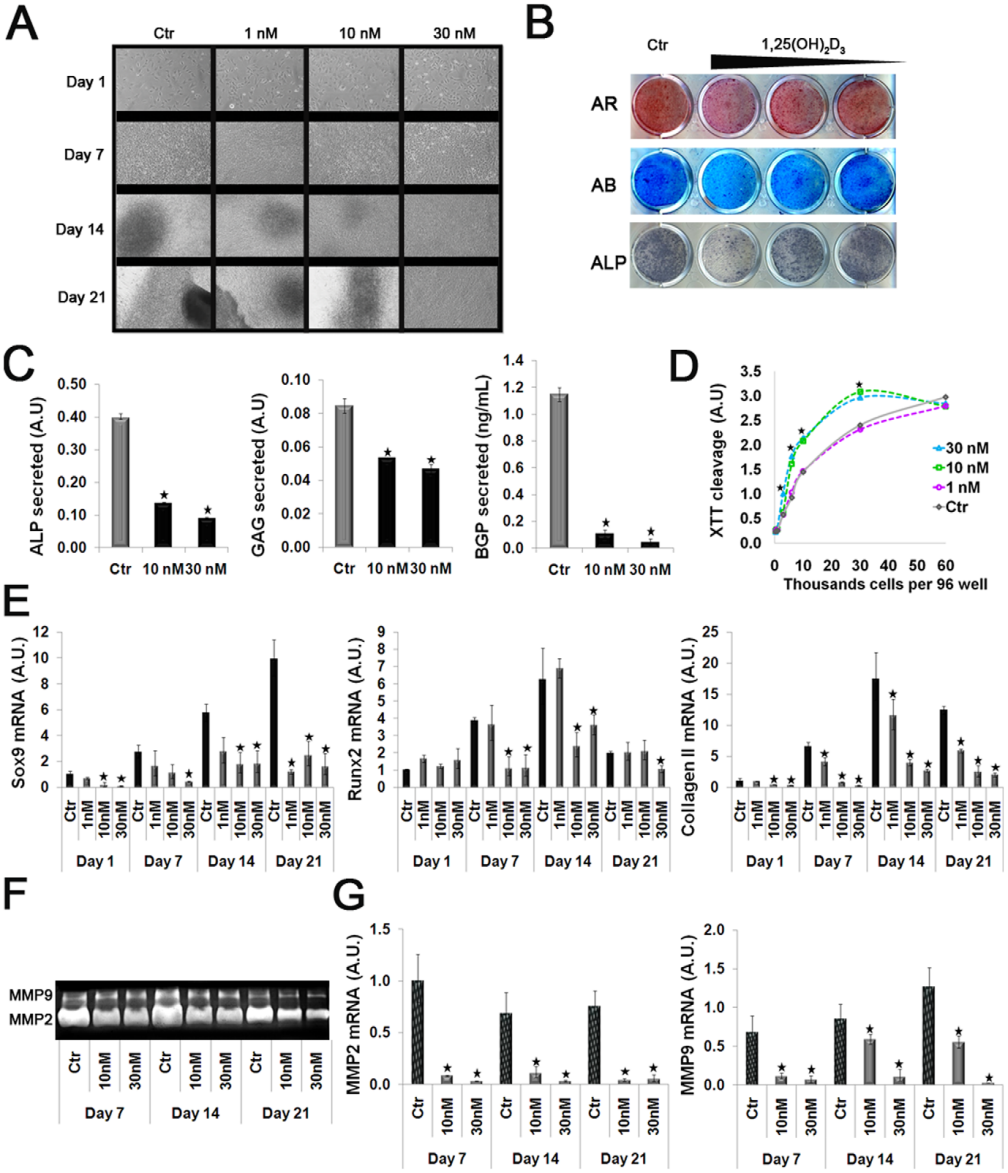
1,25(OH)_2_D_3_ dose and time-dependently suppresses chondrocytic differentiation of ATDC5 cells. ATDC5 cells were seeded at equal density (5×10^4^ cells / well in 12 well plates) and allowed to differentiate for a period of 21 days. Treatment was initiated the following day using 1, 10, 30 nM 1,25(OH)_2_D_3_ or vehicle (control) dissolved in normal medium. (A) Light microscopy of cells treated with increasing concentrations of 1,25(OH)_2_D_3_ compared to control cells over the course of differentiation. (B) Staining with Alcian Blue (for glycosaminoglycans), Alizarin Red (for mineral deposits), and ALP activity of 1,25(OH)_2_D_3_-treated compared to control cells (representative day 14 is shown). (C) Quantitative measurement of secreted ALP (pNPP hydrolysis assay), glycosaminoglycans (DMB assay), and BGP (ELISA kit) in the cell media (representative day 14 is shown). (D) Colorimetric tetrazolium salt (XTT) mitochondrial cleavage into formazan derivative assay in cells plated at indicated densities. (E) Real-time PCR analysis of chondro-osteogenic markers: Sox9, Runx2, and collagen type II normalized to GAPDH. (F) Zymography using SDS-PAGE copolymerized with gelatin, detecting MMP2 and MMP9 enzymatic activity in the media of 1,25(OH)_2_D_3_-treated compared to control cells. (G) Real-time PCR analysis of MMP2 and MMP9 normalized to GAPDH. In all studies, results are shown as means (n = 3) ± SD. * denotes p<0.05 comparing 1,25(OH)_2_D_3_-treated to control cells. Abbreviations: AU-Arbitrary Units.

The foregoing observation of compressed aggrecan, collagen type II and type X raise a possibility of direct influence of 1,25(OH)_2_D_3_ and VDR activation on chondrocytes maturation. We tested the effect of the same formulation of 1,25(OH)_2_D_3_ used in our animal studies on the chondrogenic profile of ATDC5 cell line. ATDC5 are capable of undergoing differentiation and form mineralized nodules in culture. Treatment with 1, 10, or 30 nM 1,25(OH)_2_D_3_ over the course of 21 days, inhibited the formation of mineralized nodules in a dose-dependent manner (Fig. 3A). This was accompanied by inhibited accumulation of proteoglycans (GAG, Alcian Blue staining), mineral deposition (Alizarin Red staining) and alkaline phosphatase (ALP, NBT/BCIP staining) activity (Fig. 3B). Quantitative analysis of cell media over the course of differentiation also revealed a significant reduction in the secretion of ALP, GAG and Bone Gla Protein (BGP), known to be expressed in the mature chondrocytes (Fig. 3C). Concomitant with reduced differentiation and mineralization, 1,25(OH)_2_D_3_ treatment increased the metabolic cleavage of XTT, indicative of higher cell proliferation, over a range of cell densities (Fig. 3D). mRNA levels of key transcription factors Sox 9, Runx 2, mRNA of Collagen type II, as well as mRNA and enzymatic activity of the matrix metalloproteinases MMP2 and MMP9, measured by gelatin-zymography, were all downregulated by 1,25(OH)_2_D_3_ in a dose and time dependent manner throughout differentiation (Fig. 3E, F, G). Overall, in accordance with the *in vivo* observations, these *in vitro* data point toward positive effect on the proliferation and negative effect on the maturation of chondrocytes by 1,25(OH)_2_D_3_ treatment.

### 1-week and 1-month 1,25(OH)_2_D_3_ administration reduced cortical bone thickness and elevated porosity

**Figure 4:**
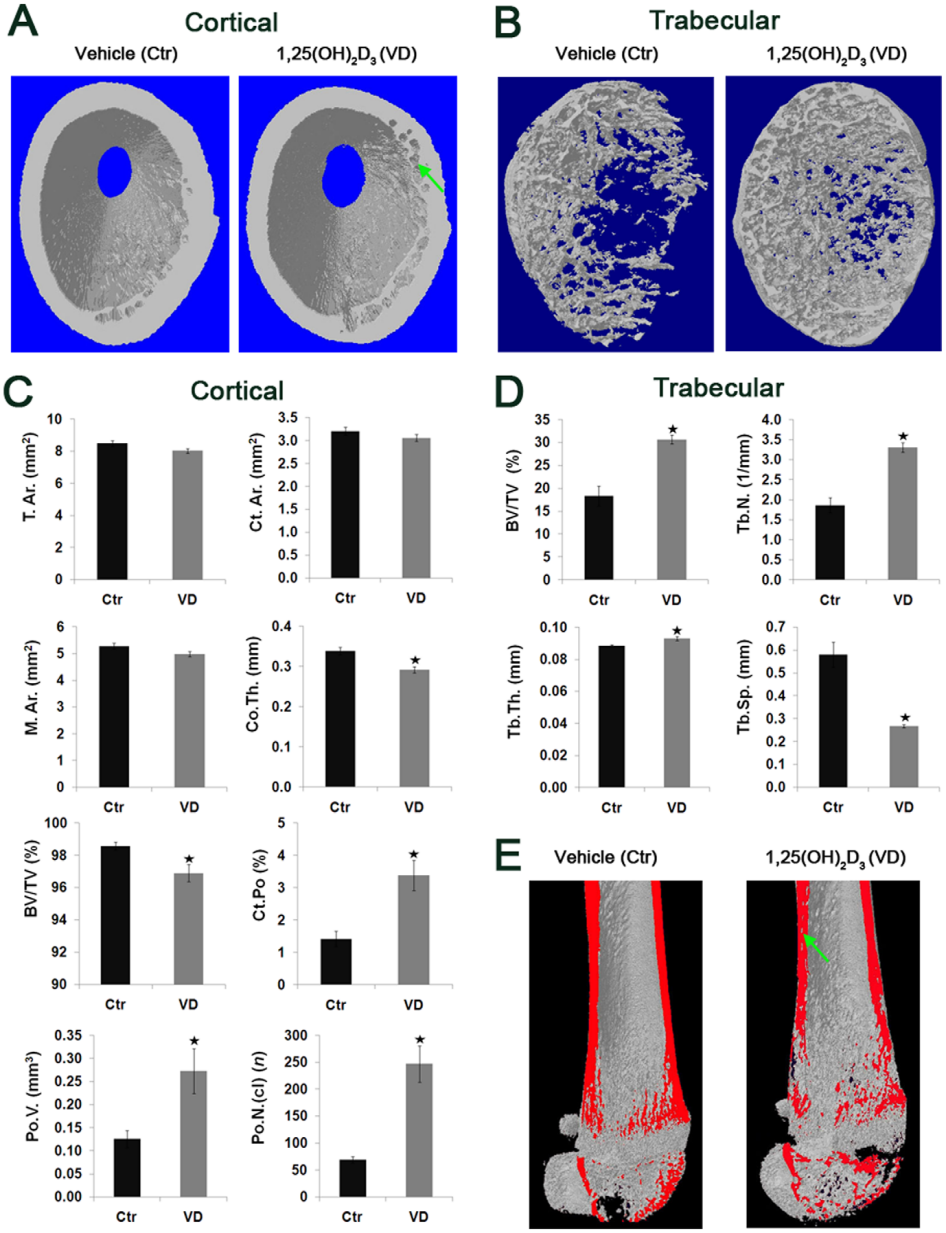
1-week administration of 1,25(OH)_2_D_3_ alters cortical bone architecture by reducing thickness and elevating porosity, and trabecular parameters by increasing number and thickness in young rats. 4 weeks old male Spague Dawley rats received daily i.p. injections of 1 µg/kg 1,25(OH)_2_D_3_ (n = 6, VD) or vehicle (n = 6, Ctr) for a period of 7 days. Femurs were removed and subjected to μ-CT scan from proximal femoral metaphysis to the mid-point of diaphysis using 13.4 µm pixel size resolution and 3500 ms laser exposure. (A) 3D images of cortical bones. Note the reduced cortical thickness and elevated porosity in the 1,25(OH)_2_D_3_-treated group, marked by green arrow. (B) 3D images of trabecular bones. Note the markedly increased trabecular number in the 1,25(OH)_2_D_3_-treated group. (C) Cortical morphological parameters: total cross-sectional area (Tt.Ar.), cortical bone area (Ct.Ar.), medullary area (M.Ar.), cortical thickness (Co.Th.), bone volume over total volume (BV/TV), percent cortical porosity (Ct.Po.), total volume of pores (Po.V.) and number of closed pores (Po.N.(cl)). (D) Trabecular bone morphological parameters: bone volume over total volume (BV/TV), trabecular number (Tb.N), trabecular thickness (Tb.Th.), trabecular separation (Tb.Sp.). Results are shown as means (n = 6) ± SE. * denotes p<0.05 comparing 1,25(OH)_2_D_3_-treated to control group. (E) 3D images of total scan cut by coronal plane (marked red). Note the higher presence of pores in the cortical bone of 1,25(OH)_2_D_3_-treated group compared to control group, marked by green arrow.

To our knowledge the effects of 1,25(OH)_2_D_3_ on young bone during rapid growth under normal physiological conditions were not previously examined using high-resolution imaging technologies. In this study we used microcomputed tomography (micro-CT) technique to evaluate whether alterations in the growth plate coincided with alterations in three-dimentional bone structure. 1-week administration of 1,25(OH)_2_D_3_ resulted in 14% lower cortical bone thickness (Co.Th.) compared to control, and the effect exacerbated to 45% reduction after 1-month 1,25(OH)_2_D_3_ treatment (Fig. 4A,C, 5A,C). No significant changes in the total cross-sectional area (T.Ar.), cortical bone area (Ct.Ar.) and medullary area (M.Ar.) were observed after 1-week administration, whereas 1-month 1,25(OH)_2_D_3_ group showed 28% reduction in Ct.Ar and 22% increase in the M.Ar, indicating thinner cortical femurs. Notably, 1,25(OH)_2_D_3_ triggered a significant reduction in bone volume over total volume (BV/TV) which fell to 7% lower than control during 1-month regimen. As BV/TV represents the relative ratio between bone and open space, the reduction BV/TV resulted in reciprocal 138% elevation in the percent cortical porosity (Ct.Po.) after 1-week (BV/TV of VD 96.9% compared to Ctr 98.6%) and 416% elevation after 1-month (BV/TV of VD 91.5% compared to Ctr 98.3%) 1,25(OH)_2_D_3_ treatment (Fig. 4C, 5C). Comparison of Ct.Po. between the bones of the control groups showed no significant differences, indicating unchanged bone volume and pore volume fractions over three week growth time. The total volume of pores increased by 117% after 1-week 1,25(OH)_2_D_3_ and by 295% after 1-month 1,25(OH)_2_D_3_, whereas the number of closed pores ((Po.N.(cl)), which accounts for small fraction of total pores, escalated from 258% to 400% higher than the respective control groups (Fig. 4C, 5C). The presence of 1,25(OH)_2_D_3_-elevated cortical pores is evident in the 3D model representations, indicated by arrows (Fig. 4A,E, 5A,E). These observations point toward a negative influence of 1,25(OH)_2_D_3_ on bone quality, starting from 1-week administration and intensifying by 1-month exposure.

**Figure 5:**
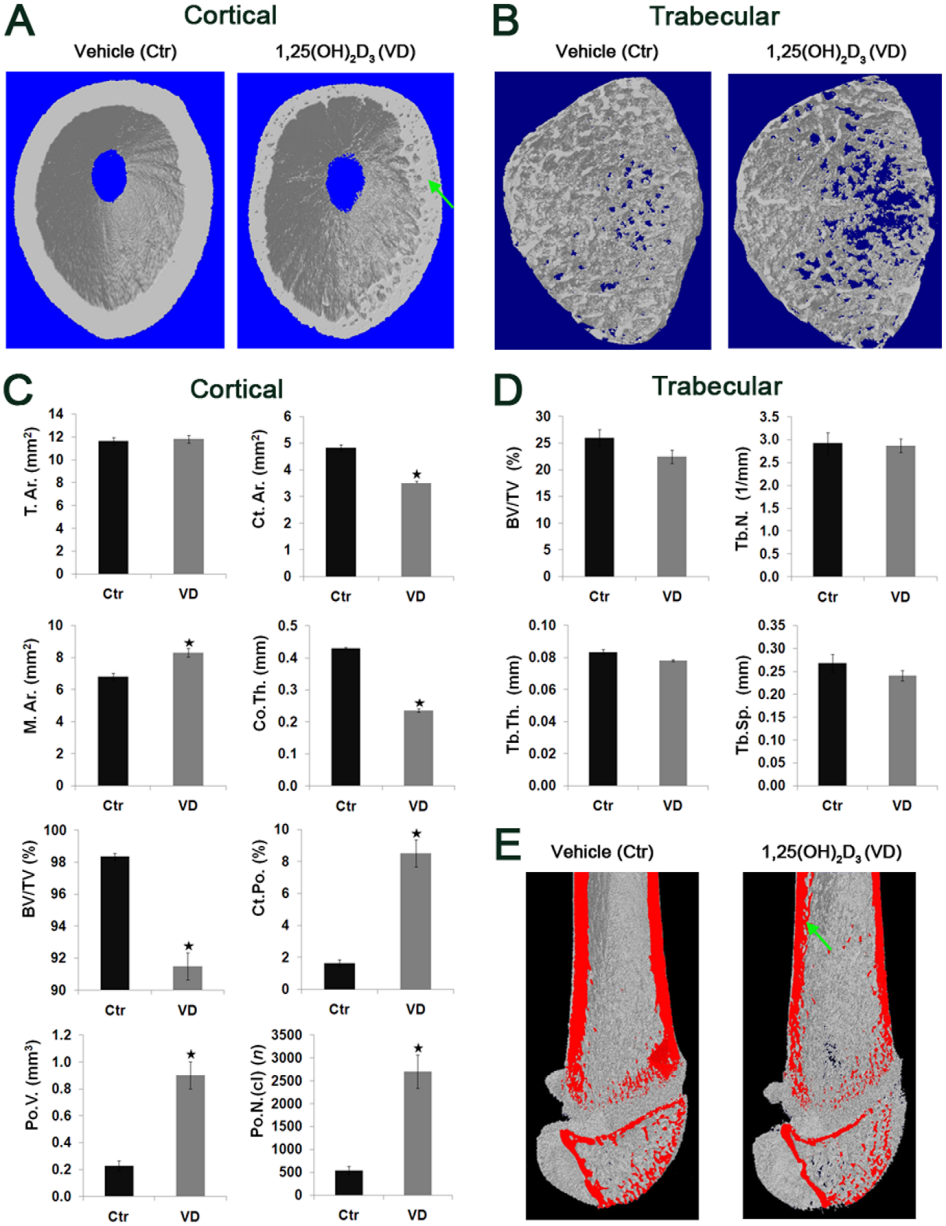
1-month administration of 1,25(OH)_2_D_3_ alters cortical bone, but not trabecular bone, architecture by reducing thickness and elevating porosity in young rats. 4 weeks old male Spague Dawley rats received i.p. injections of 3 µg/kg 1,25(OH)_2_D_3_ (n = 6, VD) or vehicle (n = 6, Ctr) on alternating days, three times a week for a period of 30 days. Femurs were removed and subjected to μ-CT scan from proximal femoral metaphysis to the mid-point of diaphysis, using 13.4 µm voxel size resolution and 4000 ms laser exposure. (A) 3D images of cortical bones. Note the reduced cortical thickness and elevated porosity in the 1,25(OH)_2_D_3_-treated group compared to control, as indicated by green arrows. (B) 3D images of trabecular bones. (C) Cortical morphological parameters. (D) Trabecular bone morphological parameters. For abbreviations see figure 4. Results are shown as means (n = 6) ± SE. * denotes p<0.05 comparing 1,25(OH)_2_D_3_-treated to control group. (E) 3D images of total scan cut by coronal plane (marked red). Note the reduced thickness and higher presence of pores in the cortical bone of 1,25(OH)_2_D_3_-treated group compared to control group, as indicated by green arrows.

### 1,25(OH)_2_D_3_ 1-week administration increases trabecular bone, which is comparable to control after 1-month treatment

1-week 1,25(OH)_2_D_3_ administration triggered significant increases of 68% in BV/TV, 77% in trabecular number (Tb.N.), and 5% in trabecular thickness (Tb.Th.), while causing a corresponding 54% reduction in the trabecular separation (Tb.Sp.) compared to control (Fig. 4B). Analysis of trabecular bone parameters in one month experiment failed to reveal any differences between 1,25(OH)_2_D_3_-treated or vehicle-treated groups (Fig. 5B). Interestingly, the 1,25(OH)_2_D_3_-induced acceleration in trabecular bone formation during 1-week treatment, resulted in bone profile similar to one month control. The fact that 1-month 1,25(OH)_2_D_3_-treatment did not further enhance bone formation, may arise from yet to be indentified, restricting mechanisms which exert negative-feedback regulation, characteristic to many endocrine systems. These results imply that 1,25(OH)_2_D_3_ enhances trabecular bone modeling, correlating to the increased apoptosis observed at the level of hypertrophic zone chondrocytes.

### 1,25(OH)_2_D_3_ inhibits cortical bone formation and mineralization

**Figure 6:**
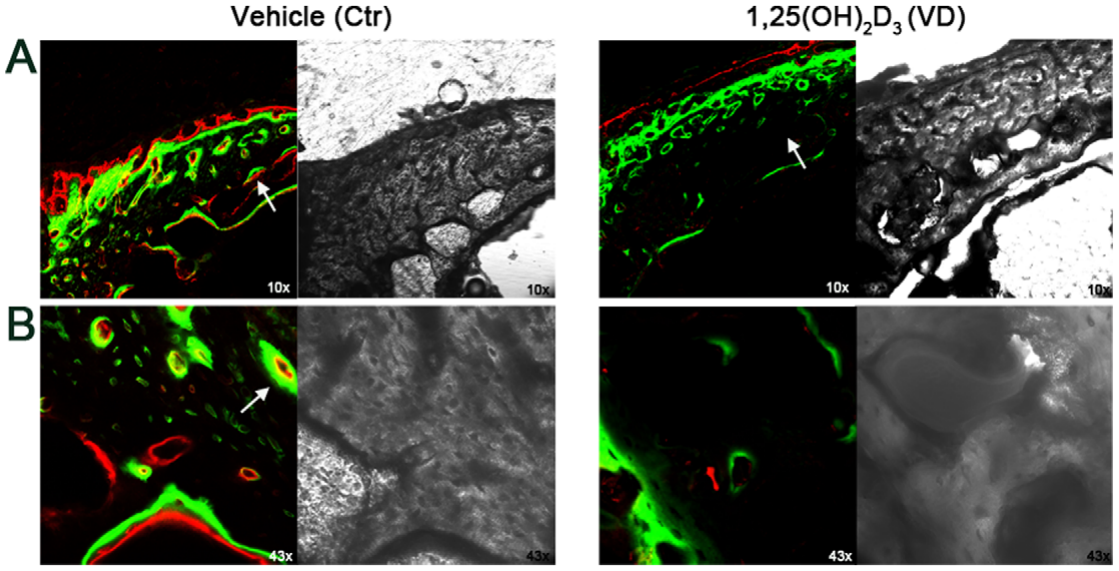
1-week administration of 1,25(OH)_2_D_3_ suppresses intracortical mineral deposition in young rats. 4 weeks old male Spague Dawley rats received daily i.p. injections of 1 µg/kg 1,25(OH)_2_D_3_ (n = 6, VD) or vehicle (n = 6, Ctr) for a period of 7 days. Both groups also received i.p. injections of mineral binding fluorochromes, 40 mg/kg calcein (green) on day 2 and 20 mg/kg alizarin complexon (red) on day 5. Femurs were removed, mid-diaphysis segments transversally cut into 200 µm sections, embedded acrylate and polished until a final thickness of 80 µm. Samples were mounted on glass slides and subjected to LSCM. (A) 10× magnification. White arrows point to the fluorochrome binding surrounding cortical pores. Note the markedly impaired mineral deposition in the 1,25(OH)_2_D_3_-treated group compared to the control group. (B) 43× magnification. Representative images from n = 3 in each group are shown.

Micro-CT analysis of cortical mineral density (BMD) demonstrated significant 10±2% and 22±2% reduction during 1-week and 1-month 1,25(OH)_2_D_3_ treatments respectively. In attempt to elucidate the reasons for 1,25(OH)_2_D_3_-increased cortical porosity and reduced BMD, we monitored the dynamics of bone formation by injecting two fluorochromes, calcein and alizarin complexone at different time points, known to bind precipitated mineral. Confocal microscopy images showed progressive time dependent bone deposition (first green calcein and subsequently red alizarine complexone) leading to pore closure in the control group (Fig. 6A,B). 1-week treatment with 1,25(OH)_2_D_3_ demonstrated markedly reduced fluorochrome staining, particularly surrounding pore area indicating hampered bone formation and thus reduced pore closure. Bone apposition at the periosteal side does not appear to be affected, supported by the unchanged T.Ar. values (Fig. 4C, 5C). These data suggest that larger percentage of pores observed during 1,25(OH)_2_D_3_ administration results from repressed bone formation in the interior and the endosteal side of the cortical bone of young growing rats.

## Discussion

To our knowledge, the current study is the first to utilize 3D imaging for presenting differential effects of supraphysiological doses of 1,25(OH)_2_D_3_ on the trabecular and cortical bone architecture in healthy young rats. We have shown that both 1-week-continuous and 1-month-intermittent administration of 1,25(OH)_2_D_3_ results in significantly narrower growth plates, influencing chondrocytes proliferation, maturation and apoptosis. Alterations in growth plate morphology coincide with changes in bone architecture, transiently elevated trabecular bone and suppressed cortical bone formation, which exacerbated with prolonged 1,25(OH)_2_D_3_ administration. Overall, our results suggest that high pharmacological doses of 1,25(OH)_2_D_3_ may directly hamper endochondral ossification and appositional bone development in the growing skeleton.

Longitudinal bone growth is largely dependent on the cartilaginous growth plates, where chondrocytes proliferate in a columnar manner, hypertrophy and undergo subsequent replacement by primary spongiosa, constituting mineralized trabecular bone. Any condition or interruption of this orderly transition can lead to bone deformities and decline the growth potential [31]. Children and animals with vitamin D deficiency or VDR mutations have expanded growth plates, known as rickets, which are largely corrected by high calcium diet [1], [3]. Aberrant growth plate, however, appears before the onset of hypocalcemia in VDR-deficient mice [34], supporting a defined role for 1,25(OH)_2_D_3_ in the process of endochondral bone formation. Recent literature supports both systemic (mineral homeostasis centered) and local (VDR-mediated activity in chondrocytes and osteoblasts) of 1,25(OH)_2_D_3_. Whereas most studies examine the effect of 1,25(OH)_2_D_3_ in transgenic models or in conjunction with either nephrectomy or ovariectomy, we tested the effect of pharmacological 1,25(OH)_2_D_3_ administration on growth plate development under normal physiological conditions. Our observations of 1,25(OH)_2_D_3_–mediated compression of hypertrophic chondrocytes, are in line with the data from VDR or 1α-hydroxylase deficient animals, where hypertrophic chondrocyte zone spreads in a rickets-like manner [3], [35]. Moreover, the restricted chondrocytes hypertrophy associated with TUNEL-detected apoptotic death in our models correlates with the above studies, where growth plate expansion was reported to be secondary to the decreased apoptosis. Although, the width of the proliferative layer was not altered, 1,25(OH)_2_D_3_ treatment caused an increase in the number of the proliferative cells both *in vivo* and in the ATDC5 chondrocytic cell line *in vitro*. On the other hand, analysis of maturation markers, including Collagen type II and X, revealed down-regulation by 1,25(OH)_2_D_3_ in the ATDC5 and compressed expression in the rat growth plates. In this regard, a collection of *in-vitro* findings previously showed differential effect of 1,25(OH)_2_D_3_ on the proliferation and differentiation of a variety of chondrocytic cell types, where the responsiveness depends on the state of maturation, dose and the time course of treatment [31], [36]. A study in uremic rats also showed that both daily and intermittent calcitriol therapy actively enhance markers of chondrocyte proliferation (cyclin D, histone-4, mTOR) and the expression of the Fibroblast Growth Factor Receptor-3 (FGFR3), a known chondrogenesis inhibitor localized to the hypertrophic zone, compared to the nephrectomized control [18]. In contrast to our observations, however, the same study reported higher expression level of VDR, Calcium Receptor (CaR), Fibroblast Growth Factor-23 (FGF-23) associated with hypertrophic chondrocytes. This discrepancy could possibly arise from the important role VDR, CaR and FGF-23 play in the maintenance of calcium and phosphorus homeostasis which is strongly perturbed in the model of renal failure. Overall, we speculate that our observations result from the direct stimulatory influence of 1,25(OH)_2_D_3_ on chondrocyte proliferation, and either an inhibitory influence on chondrocyte maturation or induction of the neighboring bone-residing osteoblasts to dominate the process of osteogenesis over chondrogenesis.

In attempt to translate the altered proliferation and differentiation from the level of growth plate to the level of bone modeling, we subjected bones to micro-CT analysis. By comparison, 1-week 1,25(OH)_2_D_3_ administration resulted in trabecular bone state analogous to the three weeks older, 1-month control group. Despite similar growth plate phenotype, 1-month 1,25(OH)_2_D_3_ administration did not further enhance trabecular bone development, possibly due to additional mechanisms controlling bone growth capacity. Such anabolic effects of active vitamin D were previously reported following 13 day infusion of 1,25(OH)_2_D_3_ to adult rats, manifested by increased trabecular osteoid mass. Moreover, in accordance with our data, histomorphometric analysis showed significantly reduced number of surface osteoclasts, suggesting lower bone resorption of the newly formed bone [37]. Additional study where high dose of 1,25(OH)_2_D_3_ was given to adult female rats resulted in augmented osteoblast recruitment and performance, reduced osteoclast number and stimulated trabecular bone remodeling [38]. Interestingly, VDR−/−, 1α-hydroxylase−/− or double mutant mice on rescue diet show reduced bone formation below wild-type, which is corrected by 1,25(OH)_2_D_3_, indicating physiological anabolic role for the endogenous 1,25(OH)_2_D_3_ mediated by VDR *in vivo* [39], [40]. Although, not tested here, the inhibitory effects of calcitriol on TRAP-positive osteoclast and chondroclast invasion may involve a mechanism of suppressed PTH release, well associated with bone resorption. Alternatively, 1,25(OH)_2_D_3_ may directly influence osteoclasts, since *ex vivo* studies reported a calcitriol VDR-mediated suppression of osteoclast differentiation from precursor cells, dependent on c-Fos protein reduction [41]. Additional supporting evidence relates to the impaired chondrocytes activity, which was previously shown to locally regulate osteoclastogenesis during bone formation [7]. Collectively, our results suggest that 1,25(OH)_2_D_3_-mediated increase in chondrocyte proliferation together with compressed maturation and enhanced apoptosis, indicate accelerated process of growth plate transition which ultimately results in increased osteogenesis.

A particularly interesting observation came from the analysis of cortical bone which in contrast to trabecular bone, portrayed a reduced osteogenesis profile, in terms of both architecture and mineral content. Separate from the longitudinal bone growth, appositional bone growth relies on the process of modeling where endosteal osteoclast resorb inner cortex surface and periosteal osteoblasts deposit and mineralize primary bone matrix on the outer perimeter [42]. Our results demonstrated that continuous or intermittent treatment with 1,25(OH)_2_D_3_ reduce cortical modeling, leaving bones 14% and 45% thinner after 1-week and 1-month treatment respectively. Increased medullar area in conjunction with normal cortical perimeter point toward uncoupled bone formation and resorption, plausibly stemming from enhanced osteoclastic activity at the endosteal surface. Previous work demonstrated that pharmacological, but not physiological doses of 1,25(OH)_2_D_3_ markedly increase RANKL expression by osteoblast, stimulating cortical osteoclastogenesis and bone resorption in rats [43]. Moreover, daily calcitriol injection in uremic rats was reported to result in lower cortical thickness, compared to nephrectomized control [20]. These data partially support the fact that cortical bone is affected more dramatically during renal failure than trabecular bone, in the pediatric patients on peritoneal dialysis and calcitriol therapy [9]. Inside the cortex, the coupling between osteoblast and osteoclast activity is manifested in the presence of cortical pores which provide conduits for the passage of neurovascular structures. Cortical porosity is considered to be closely associated with mechanical properties of cortical bone, influencing bone strength and risk of fracture. We showed that 1-week 1,25(OH)_2_D_3_ administration dramatically augmented the volume and number of cortical pores, which exacerbated to over 4 fold higher percent cortical porosity in relation to control upon 1-month administration. Since larger medular cavity implies enhanced osteoclast presence, we speculate that elevated bone resoprtion, either alone or in combination with suppressed osteoblast function, accounts for higher porosity. A recent notion of two distinct osteoblast types situated in the cortical bone [44], raises another possibility, whereby 1,25(OH)_2_D_3_ selectively suppresses the intracortical osteoblasts lining primary osteons, without affecting surface mesenchymal osteoblasts lining the periosteum. Finally, analysis of mineral binding flurochromes demonstrated that administration of 1,25(OH)_2_D_3_ dramatically hampered intracortical collagen matrix deposition and mineralization leading to the deficient filling of pores. Previous literature reports using classical 2D histomorphometric methods show inhibitory effect of pharmacological dose of 1,25(OH)_2_D_3_ on cortical bone remodeling in ribs of adult dogs [45] and impaired mineralization in adult rats [37]. Our observations suggest that although osteoblast-dependent radial cortical growth was not affected, 1,25(OH)_2_D_3_ augmented endosteal osteolclast activity and largely disturbed intracortical bone remodeling and mineralization.

To offset the 1,25(OH)_2_D_3_-related ABD and cardiovascular components of CKD, novel less hypercalcemic analogues of vitamin D, including paricalcitol and doxercalciferol, have already been available for use in adult patients [10]. In light of the significant adverse effects of calcitriol, search for alternative treatments in CKD-MBD which ensure optimal growth plate physiology and resolution of secondary hyperparathyroidism without the burden of systemic vasculopathy are in place.

## Supporting Information

Figure S1 **1,25(OH)_2_D_3_ induces vascular calcification in the aortae of young rats.** 4 weeks old male Spague Dawley rats were treated with either 1 µg/kg 1,25(OH)_2_D_3_ (n = 6, VD) for a period of 1 week or with 3 µg/kg 1,25(OH)_2_D_3_ (n = 6, VD) three times a week for a period of 1 month. Control groups were treated according to the same respective schedules with vehicle (n = 6 in each control group). Descending aortae were embedded in paraffin blocks, cut into 5 µm sections and subjected to histological analysis. (A) Alizarin Red (stain for mineral deposition) of aortae in 1 week 1,25(OH)_2_D_3_ experiment. (B) Alizarin Red staining of aortae in 1 month 1,25(OH)_2_D_3_ experiment. Abbreviations: L-lumen, M-media, A-adventitia. Green arrows point toward the diffuse calcification of the aortic media and the neighboring blood vessel. Note that adventitia does not stain red and remains unmineralized.
(TIF)
