# Supplementary figures and images for: 1,25(OH)2D3 Alters Growth Plate Maturation and Bone Architecture in Young Rats with Normal Renal Function
Source: PLoS One. 2011 Jun 13;6(6):e20772. doi: 10.1371/journal.pone.0020772 (PMC3113808; doi:10.1371/journal.pone.0020772)

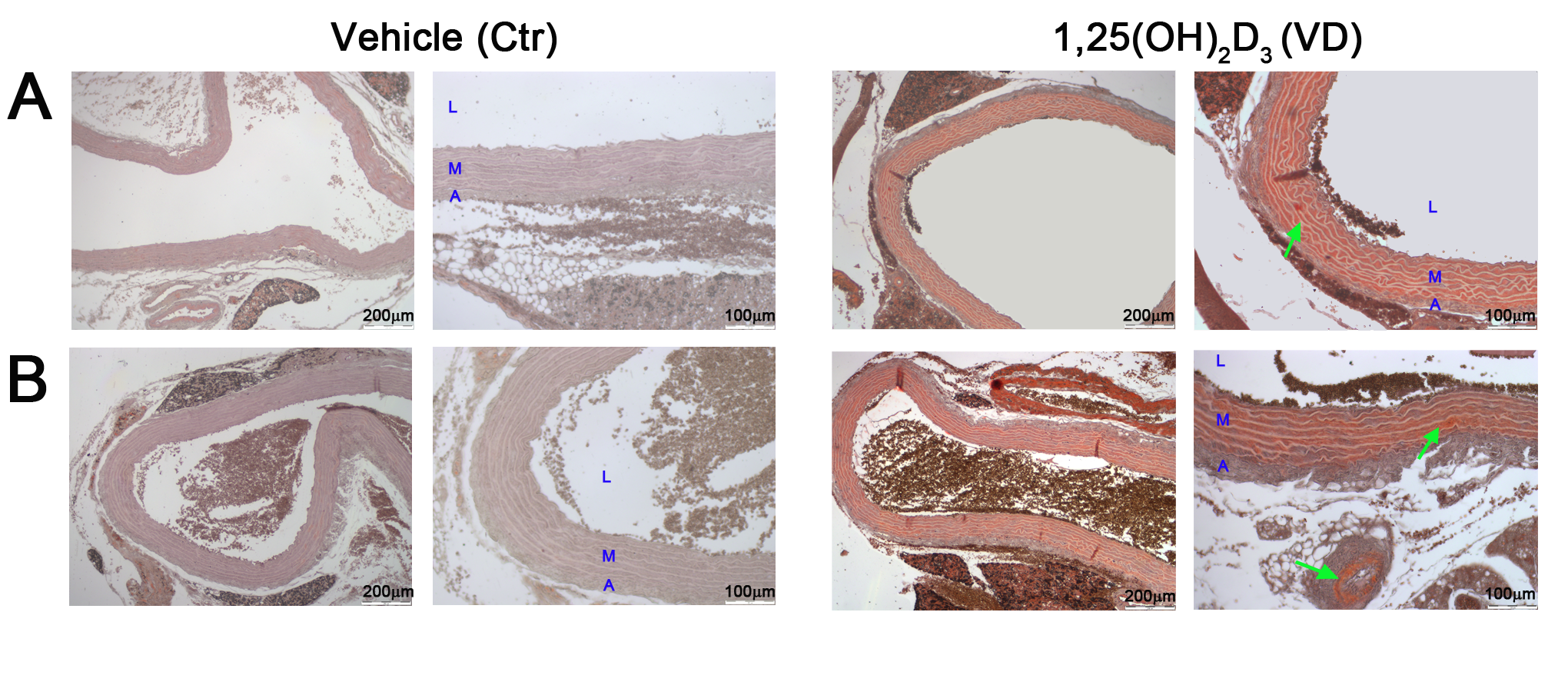

Supplement: Figure S1 — 1,25(OH)2D3 induces vascular calcification in the aortae of young rats. 4 weeks old male Spague Dawley rats were treated with either 1 µg/kg 1,25(OH)2D3 (n = 6, VD) for a period of 1 week or with 3 µg/kg 1,25(OH)2D3 (n = 6, VD) three times a week for a period of 1 month. Control groups were treated according to the same respective schedules with vehicle (n = 6 in each control group). Descending aortae were embedded in paraffin blocks, cut into 5 µm sections and subjected to histological analysis. (A) Alizarin Red (stain for mineral deposition) of aortae in 1 week 1,25(OH)2D3 experiment. (B) Alizarin Red staining of aortae in 1 month 1,25(OH)2D3 experiment. Abbreviations: L-lumen, M-media, A-adventitia. Green arrows point toward the diffuse calcification of the aortic media and the neighboring blood vessel. Note that adventitia does not stain red and remains unmineralized. (TIF) [file pone.0020772.s001.tif]
